# ﻿*Poxyaibamberus* Andersen & Dantas, gen. nov. (Diptera, Chironomidae, Orthocladiinae) from Brazil

**DOI:** 10.3897/zookeys.1205.124081

**Published:** 2024-06-14

**Authors:** Trond Andersen, Galileu P. S. Dantas, Viktor Baranov, Annui M. Sanz-laParra, Humberto F. Mendes, Neusa Hamada

**Affiliations:** 1 Department of Natural History, University Museum of Bergen, University of Bergen, P.O. Box 7800, NO-5020 Bergen, Norway University of Bergen Bergen Norway; 2 Instituto Nacional de Pesquisas da Amazônia, Coordenação de Biodiversidade (CoBio), PPG_ENTO, Av. André Araújo, 2936, 69067–375, Manaus, Amazonas, Brazil Instituto Nacional de Pesquisas da Amazônia, Coordenação de Biodiversidade (CoBio) Manaus Brazil; 3 Estación Biológica de Doñana-CSIC / Doñana Biological Station-CSIC, C. Américo Vespucio, s/n, 41092 Seville, Spain Estación Biológica de Doñana-CSIC / Doñana Biological Station-CSIC Seville Spain; 4 Department of Biology, Instituto de Ciências da Natureza, Universidade Federal de Alfenas, Rua Gabriel Monteiro da Silva, 700, 37130-001 Alfenas, Mato Grosso, Brazil Universidade Federal de Alfenas Alfenas Brazil

**Keywords:** Aquatic insects, new species, Neotropical Region, taxonomy

## Abstract

*Poxyaibamberus* Andersen & Dantas, **gen. nov.** is erected based on the males of two species, *P.jamanximensis* Andersen & Dantas, **sp. nov.** from Jamanxim National Park, Pará State, Brazil, and *P.ubajarensis* Andersen & Dantas, **sp. nov.** from Ubajara National Park, Ceará State, Brazil. Both species have a comparatively short and wide head, with large eyes and short, five-segmented palps; a strong subapical seta on the ultimate flagellomere; scalpellate acrostichals; no setae on the wing veins except for one seta on the brachiolum; a long costal extension; and a large triangular anal point and a very long heel on the gonostylus. The systematic position of the new genus is briefly discussed.

## ﻿Introduction

The number of orthoclad species known from Brazil has increased rapidly during the last three decades. In their catalog of the Neotropical and Mexican chironomids Spies and Reiss (1996) recorded eight Orthocladiinae species in six genera from Brazil; one of these, *Ichthyocladiusneotropicus* Fittkau, 1974, was listed as uncertain and has later been proven to not occur in Brazil (Mendes et al. 2004). Today more than 144 species in 44 genera are known to occur in the country (Pinho et al. 2024), although there is still a significant gap in the North and Northeast regions, where only 23 and 14 species of Orthocladiinae have been recorded. Many orthoclads encountered in Brazil do not readily fit into any described genus, and many new genera have been described. Several genera, namely *Gynocladius* Mendes, Sæther & Andrade-Morraye, 2005; *Oleia* Andersen & Mendes, 2007; *Saetherocladius* Andersen & Mendes, 2007; *Saetherocryptus* Andersen & Mendes, 2007; *Saetherolabis* Andersen & Mendes, 2007; *Saetherops* Andersen & Mendes, 2007; *Lyrocladius* Mendes & Andersen, 2008; *Ubatubaneura* Wiedenbrug & Trivinho-Strixino, 2009; *Iporangomberus* Mendes & Andersen, 2012; *Pebapomberus* Mendes & Andersen, 2012; *Miambera* Andersen & Mendes, 2012; *Maximberus* Andersen & Mendes, 2012; *Jururumberus* Mendes & Andersen, 2013; *Uirassubrillia* Mendes, Andersen & Pinho, 2013; *Caaporangombera* Andersen, Pinho & Mendes, 2015; *Mariambera* Andersen, Mendes & Pinho, 2015; and *Urubicimbera* Andersen, Pinho & Mendes, 2015 are so far endemic to Brazil (Mendes et al. 2005, 2013; Andersen and Mendes 2007, 2012a, 2012b; Mendes and Andersen 2008, 2012a, 2012b, 2013; Wiedenbrug and Trivinho-Strixino 2009; Andersen et al. 2015a, 2015b, 2015c). However, several of these genera are expected to be more widely distributed when the chironomid fauna of neighboring countries is better studied.

Below we describe a new genus based on the males of two new species collected in Pará and Ceará states in northern and northeastern Brazil. Both species have a comparatively wide head with large eyes and short, five-segmented palps, a strong subapical seta on the ultimate flagellomere, scalpellate acrostichals, a long costal extension, no setae on the wing membrane and veins except for one seta on the brachiolum, a large triangular anal point, and a gonostylus with a very long heel. The systematic position of the new genus is briefly discussed.

## ﻿Materials and methods

The specimens were collected with Shannon traps (Shannon 1939) or light traps and preserved in 80% ethanol during the fieldwork. Prior to examination they were mounted in Euparal following the procedure outlined by Sæther (1969). Morphological terminology follows Sæther (1980). Coloration is based on the slide mounted specimen.

For the phylogenetic analysis we have used a morphological character matrix with 45 taxa and 83 characters. Characters were sampled from larvae, pupae, and adult males and females. Fossils were of course underrepresented in terms of characters available for observation (see https://github.com/chironomus/Poxyaibamberus-). The character matrix for the phylogenetic analysis was built using NEXUS DATA EDITOR v. 0.5. 0 (Page 2001).

First, a Bayesian analysis of the morphological matrix alone in MRBAYES 3.2.2. (Ronquist et al. 2012) was conducted using the Bayesian implementation of Lewis’ Markov models (Lewis 2001). In Bayesian inference, two Markov chains were run simultaneously for 10 million generations using a discrete Dirichlet distribution with equal state frequencies (Lewis 2001). Substitution model was set to “gamma” (Nylander et al. 2004). The first 25 0000 generations were discarded as a burn-in (number of MRBAYES generations of the tree topology before the apparent stationary condition) (Nylander et al. 2004). Consensus trees showing all compatible groups and 50% compatible groups were computed in MRBAYES. Ancestral character state analysis was conducted on the allcompat consensus tree based on morphology alone using ANCTRESH in the PHYTOOLS package v. 0.7-80 (Revell 2012). R code for this analysis, alongside the data is provided in https://github.com/chironomus/Poxyaibamberus-.

To deal with the uncertainty of the positions of the genus on the tree caused by the lack of knowledge of character states, the Klopfstein and Spasojevic ROGUEPLOTS approach was applied (Klopfstein and Spasojevic 2019). A morphological allcompat tree from MRBAYES (as described above) was used to place *Poxyaibamberus* into the high posterior probability regions. ROGUEPLOTS for every species and accompanying R code are provided in the https://github.com/chironomus/Poxyaibamberus-.

Both holotypes are kept in the Invertebrate collection at the Instituto Nacional de Pesquisas da Amazônia (**INPA**), Manaus, Brazil.

## ﻿Taxonomic account

**Family Chironomidae Newman, 1834**

**Subfamily Orthocladiinae Kieffer, 1911**

### ﻿Genus Poxyaibamberus

Taxon classificationAnimaliaDipteraChironomidae

Andersen & Dantas gen. nov.

810CEFBD-430B-5609-8678-0CA571D40BF4

https://zoobank.org/2FE0E263-AB8C-4240-8CB7-9FF8AF3907A4

#### Type species.

*Poxyaibamberusjamanximensis* Andersen & Dantas, sp. nov.

#### Diagnosis.

Small species, wing length 1.1–1.3 mm.

#### Description.

***Male antenna*** with 13 flagellomeres, strongly plumose, groove beginning on flagellomere 4, few sensilla chaetica apparently only present on flagellomere 13, with strong subapical seta. Antennal ratio 0.9.

***Head*** short and wide. Eye bare, large, reniform, without dorsomedian extension. Temporal setae in single row, consisting of inner and outer verticals. Frontal tubercle absent. Tentorium and stipes normal. Clypeus with few setae. Palp short, with 5 segments, third palpomere without sensilla clavata subapically.

***Thorax*.** Antepronotum well developed, with lobes meeting medially at anterior margin of scutum, with few ventrolateral antepronotals. Acrostichals small, scalpellate, in two rows starting some distance from antepronotum; dorsocentrals simple, uniserial; prealars few; supraalar absent. Scutellum apparently without setae.

***Wing*.** Membrane without setae, with fine punctuation. Anal lobe reduced. With long costal extension. R_2+3_ running and ending close to R_4+5_; R_4+5_ ending distal to M_3+4_; FCu far distal to RM; Cu_1_ curved. Brachiolum with 1 seta, other veins bare. Squama bare. Sensilla campaniformia 3 above seta on brachiolum.

***Legs*.** Tibial spurs normal, comb with few setae. Pseudospurs and sensilla chaetica absent, pulvilli vestigial.

***Abdomen*.** Tergites with few setae, mostly in anterior band; sternites with few setae.

***Hypopygium*.** Anal point long, triangular, with microtrichia, with or without strong setae at base, originating high on tergite IX or at posterior margin. Tergite IX with or without setae; laterosternite IX with few setae. Phallapodeme with aedeagal lobe well developed. Transverse sternapodeme arched or straight, without oral projections. Virga apparently consists of small spines. Gonocoxite long, without volsellae. Gonostylus without crista dorsalis, with long, weakly to strongly curved heel; megaseta normal.

#### Etymology.

From Tupi *Poxyaiba*, ugly and *Mberu*, fly, meaning “the ugly fly”, referring to the spiny hypopygium of *P.jamanximensis* Andersen & Dantas, sp. nov. with a very large anal point. The name is masculine.

### ﻿ Poxyaibamberus jamanximensis

Taxon classificationAnimaliaDipteraChironomidae

Andersen & Dantas sp. nov.

E18ECE1A-0EFB-58C8-833A-4B80D0DDF665

https://zoobank.org/AC19B7F6-BE94-4368-B494-44A65957202E

Figs 1A, B , 2 , 3

#### Type locality.

Brazil, Pará State, Itaituba, Jamanxim National Park; 05°41'58"S, 55°48'13"W; 170 m a.s.l.; 20 November 2017; Gilberto Nicácio leg.

#### Type specimen.

***Holotype*** male adult, slide-mounted in Euparal under six coverslips. Original label: “Brasil, PA, 20/11/2017, Floresta # 41, Shannon trap, Orthocladiinae, leg. G. Nicasio, ♂”. (INPA).

#### Diagnostic characters.

The new species can easily be separated from *P.ubajarensis* Andersen & Dantas, sp. nov. by the shape of the gonostylus, as it has a rather narrow, weakly curved, tapering heel that is slightly longer than the gonostylus proper.

#### Description.

**Adult male** (*n* = 1). Total length 2.17 mm. Wing length 1.23 mm. Total length / wing length 1.77. Wing length / length of profemur 2.51.

***Coloration*.** Head, thorax, and legs light yellowish brown; abdomen pale yellowish brown. Wing hyaline.

**Figure 1.**
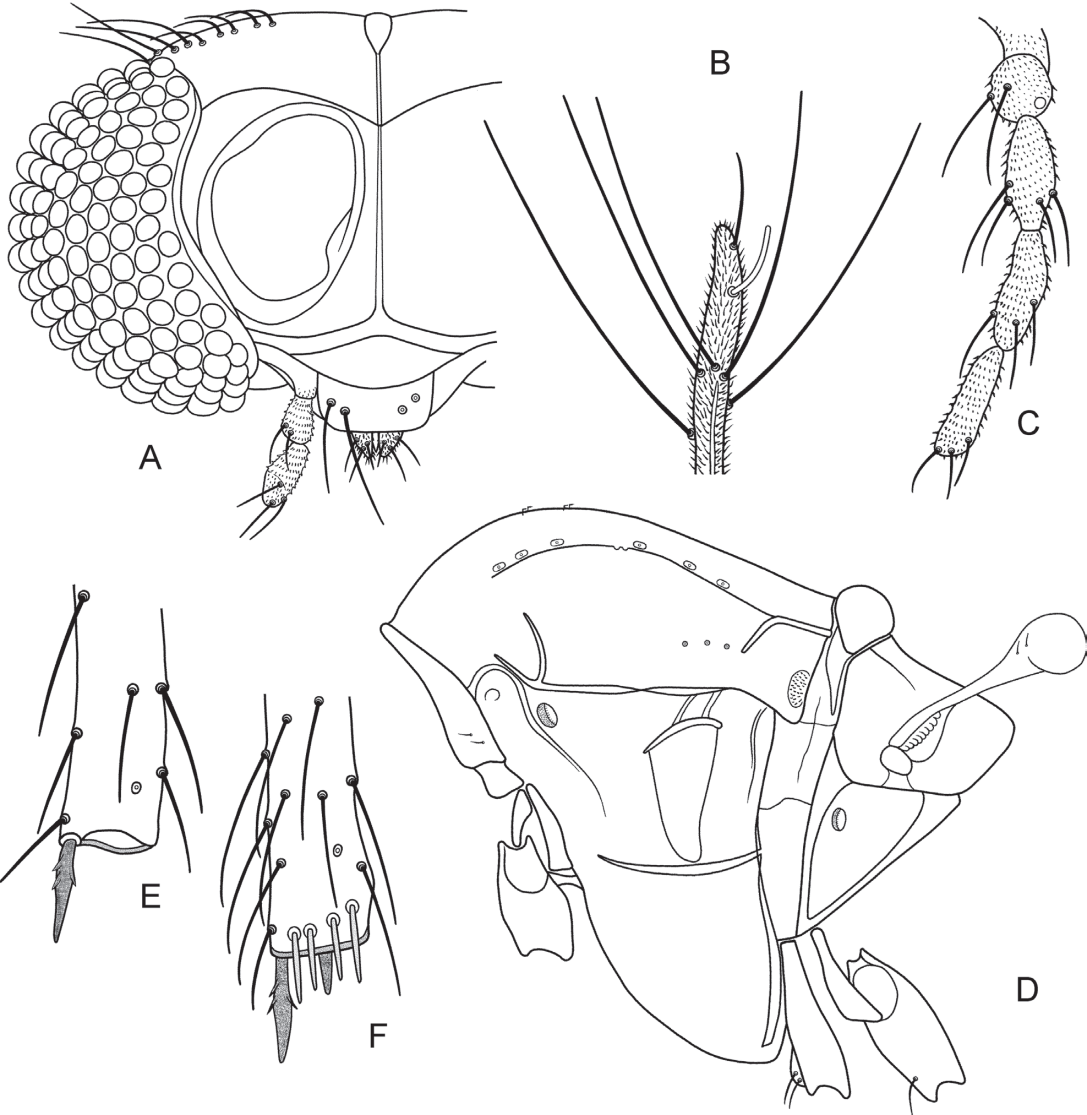
*Poxyaibamberusjamanximensis* Andersen & Dantas, sp. nov. male (**A, B**) and *P.ubajarensis* Andersen & Dantas, sp. nov., male (**C–F**) **A** head, palpomere 4 and 5 not drawn **B** apex of ultimate flagellomere **C** palp **D** thorax **E** apex of fore tibia **F** apex of hind tibia.

***Antenna*** (Fig. 1B). With 13 segments. AR = 0.94. Terminal flagellomere 340 µm long, with 21 µm long subapical seta.

***Head*** (Fig. 1A). Inner verticals 5, outer verticals 4, postorbitals not discernable. Clypeus with 4 setae. Tentorium 99 µm long, 17 µm wide. Stipes 76 µm long. Anterior margin of cibarial pump slightly convex. Palp with palpomere 4 and 5 not measurable; palpomere 1–3 lengths (in µm) as: 14, 25, 38. Third palpomere without sensilla clavata.

***Thorax*.** Antepronotum with 2 ventrolateral setae. Acrostichals about 8, scalpellate, in double row starting some distance from antepronotum; dorsocentrals 8, uniserial; prealars 3. Scutellum apparently without setae.

**Figure 2.**
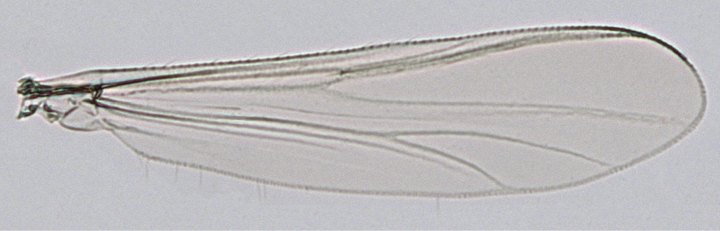
*Poxyaibamberusjamanximensis* Andersen & Dantas, sp. nov. male. Wing.

***Wing*** (Fig. 2). VR = 1.33. Brachiolum with 1 seta, other veins and membrane bare. Squama bare. Costal extension 123 µm long.

***Legs*.** Fore tibia with 33 µm long spur, mid tibia with 28 and 15 µm long spurs, hind tibia with 45 and 21 µm long spurs. Width at apex of fore tibia 27 µm, of mid tibia 28 µm, of hind tibia 29 µm. Hind tibia with comb of 4 bristles, longest apparently about 28 µm long. Lengths and proportions of legs as in Table 1.

**Table 1.** Lengths (in µm) and proportions of legs of *Poxyaibamberusjamanximensis* Andersen & Dantas, sp. nov., male (*n* = 1).

	Fe	Ti	ta_1_	ta_2_	ta_3_	ta_4_	ta_5_	LR	BV	SV	BR
**P_1_**	490	596	547	270	155	82	49	0.918	2.941	1.985	2.10
**P_2_**	564	572	343	172	98	49	33	0.600	4.209	3.310	2.30
**P_3_**	605	629	417	212	163	82	41	0.662	3.311	2.961	2.40

**Figure 3.**
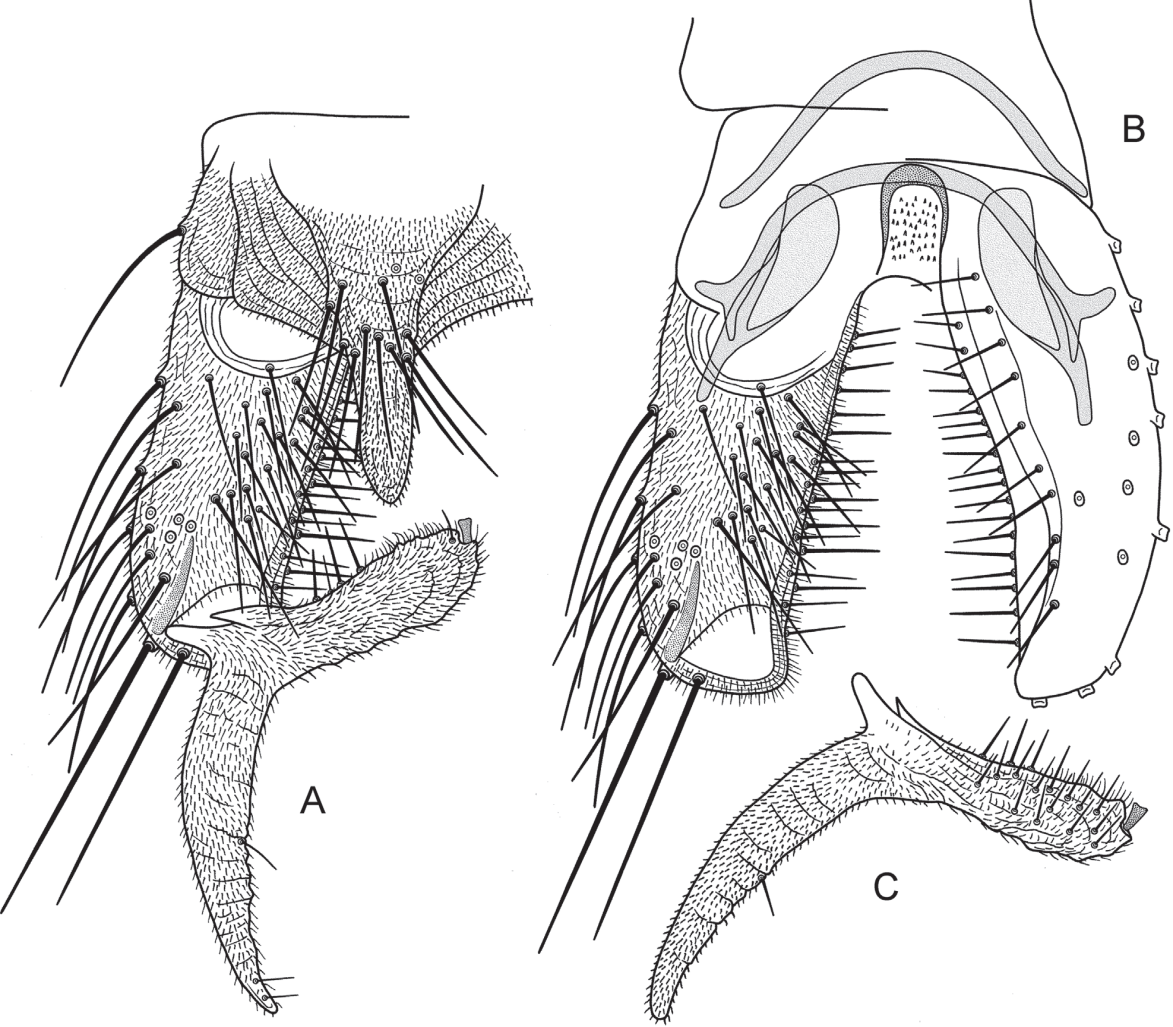
*Poxyaibamberusjamanximensis* Andersen & Dantas, sp. nov. male. **A** hypopygium, dorsal view **B** hypopygium with tergite IX and anal point removed, left dorsal aspect, right ventral aspect **C** gonostylus, ventral view.

***Hypopygium*** (Fig. 3A–C). Anal point large, broadly triangular with rounded apex, starting high on tergite IX, with microtrichia and 12 strong setae in basal 1/3; 76 µm long, 39 µm wide near base, 18 µm wide medially. Laterosternite IX with 1 seta. Phallapodeme 76 µm long. Transverse sternapodeme arched, without oral projections, 62 µm long. Virga apparently consisting of field with small spines. Gonocoxite 203 µm long. Gonostylus straight, 104 µm long; heel weakly curved, tapering, 119 µm long; megaseta 7 µm long. HR = 1.95; HV = 2.09.

**Immatures and female.** Larva, pupa, and female are unknown.

#### Etymology.

The epithet, *jamanximensis*, is used as an adjective and meaning “from Jamanxim” in reference to the place of origin of the holotype.

#### Distribution.

The species is only known from the type locality in Jamanxim National Park, Pará State, in northern Brazil, where it was collected in a Shannon trap placed near a lower-order stream. Only a single male was collected despite intensive sampling effort during the expedition with successive sampling using several types of traps. According to data from the National Institute for Space Research, the Jamanxim National Park ranks among the conservation units with the highest deforestation rates in the Amazon. This situation underscores the need to increase the knowledge of the biodiversity in this region to better understand the anthropogenic impacts on the biota and to utilize this knowledge as tools for developing conservation strategies. The discovery of a new genus of Chironomidae in the park highlights the importance of further research to understand and preserve its unique biodiversity.

### ﻿ Poxyaibamberus ubajarensis

Taxon classificationAnimaliaDipteraChironomidae

Andersen & Dantas sp. nov.

CB948E97-844B-5E4D-A89E-1A38ED3D6AEC

https://zoobank.org/E8261BAB-A560-419E-AF25-4951A909D760

Figs 1C–F , 4

#### Type locality.

Brazil, Ceará State, Ubajara, Ubajara National Park, Cafundó waterfall, 03°50'13"S, 40°54'35"W, 805 m a.s.l., 25–26 February 2023, N. Hamada, J. Silva, J.M.C. Nascimento, G.P. Amorim Jr. leg.

#### Type specimen.

***Holotype*** male adult, slide-mounted in Euparal under five coverslips. Original label: “Brasil, CE, Ubajara, Parque Nacional de Ubajara, Cachoeira do Cafundó, 25-26/02/2023, light-trap, N. Hamada” (INPA).

#### Diagnostic characters.

The new species can easily be separated from *P.jamanximensis* Andersen & Dantas, sp. nov. on the shape of the gonostylus, as it has a rather broad, curved, tapering heel that is slightly shorter than the gonostylus proper.

#### Description.

**Adult male** (*n* = 1). Total length 2.02 mm. Wing length 1.13 mm. Total length / wing length 1.70. Wing length / length of profemur 2.38.

***Coloration*.** Head, thorax, and legs light brown; abdomen pale brown. Wing hyaline.

***Antenna*.** Antenna broken. With 44 µm long subapical setae.

***Head*.** Inner verticals 5, outer verticals 3, postorbitals not discernable. Clypeus with 5 setae. Tentorium 90 µm long, 13 µm wide. Stipes not discernable. Anterior margin of cibarial pump slightly concave. Palp (Fig. 1C) with five segments; palpomere lengths (in µm): 17, 22, 35, 39, 37. Third palpomere without sensilla clavata.

***Thorax*** (Fig. 1D). Antepronotum with 2 ventrolateral setae. Acrostichals very small, at least 4 in double row in anterior part of scutum; dorsocentrals 6, uniserial; prealars 3. Scutellum apparently without setae.

***Wing*.** VR = 1.33. Brachiolum with 1 seta, other veins and membrane bare. Squama bare. Costal extension 113 µm long.

***Legs*.** Fore tibia (Fig. 1E) with 30 µm long spur, mid tibia with 29 µm and 14 µm long spurs, hind tibia (Fig. 1F) with 35 µm and 18 µm long spurs. Width at apex of fore tibia 28 µm, of mid tibia 29 µm, of hind tibia 30 µm. Hind tibia with comb of 4 bristles, longest 22 µm long. Lengths and proportions of legs as in Table 2.

**Table 2.** Lengths (in µm) and proportions of legs of *Poxyaibamberusubajarensis* Andersen & Dantas, sp. nov., male (*n* = 1).

	Fe	Ti	ta_1_	ta_2_	ta_3_	ta_4_	ta_5_	LR	BV	SV	BR
**P_1_**	474	507	–	–	–	–	–	–	–	–	–
**P_2_**	507	515	310	155	98	53	45	0.602	3.795	3.297	1.92
**P_3_**	605	629	–	–	–	–	–	–	–	–	–

**Figure 4.**
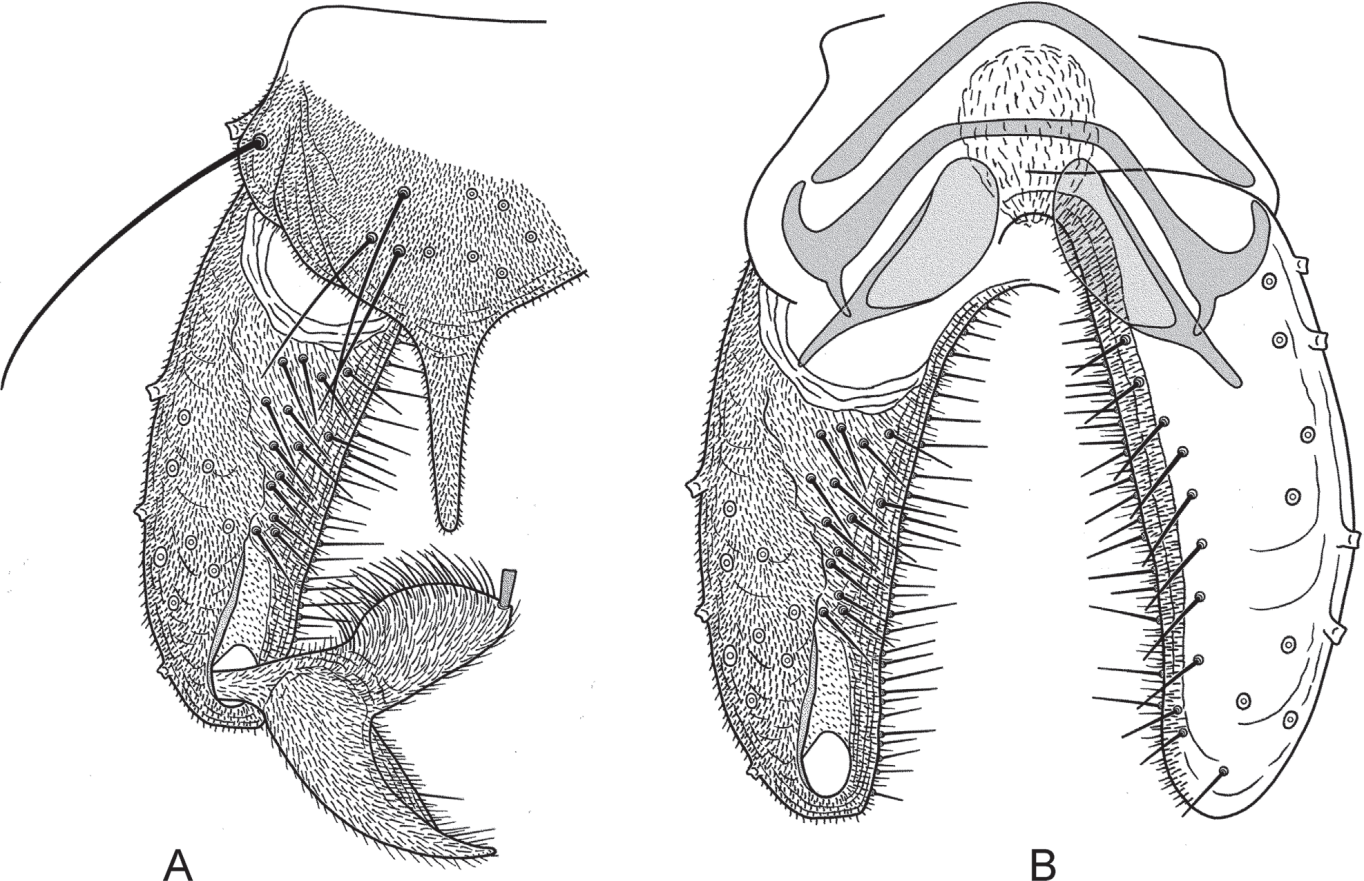
*Poxyaibamberusubajarensis* Andersen & Dantas, sp. nov. male **A** hypopygium, dorsal view **B** hypopygium with tergite IX and anal point removed, left dorsal aspect, right ventral aspect.

***Hypopygium*** (Fig. 4A, B). Anal point large, narrowly triangular with rounded apex, projecting from posterior margin of tergite IX, with microtrichia, 55 µm long, 28 µm wide near base, 8 µm wide medially. Tergite IX with 9 setae medially. Laterosternite IX with 2 setae. Phallapodeme 68 µm long. Transverse sternapodeme straight, without oral projections, 41 µm long. Virga apparently consisting of balloon-shaped ball of lamella with small spines. Gonocoxite 168 µm long. Gonostylus 79 µm long; heel curved, tapering, 72 µm long, 24 µm wide medially; megaseta 8 µm long. HR = 2.13; HV = 2.56.

**Immatures and female.** Larva, pupa, and female are unknown.

#### Etymology.

The epithet, *ubajarensis*, is used as an adjective and meaning “from Ubajara” in reference to the place of origin of the holotype.

#### Distribution.

**Figure 5.**
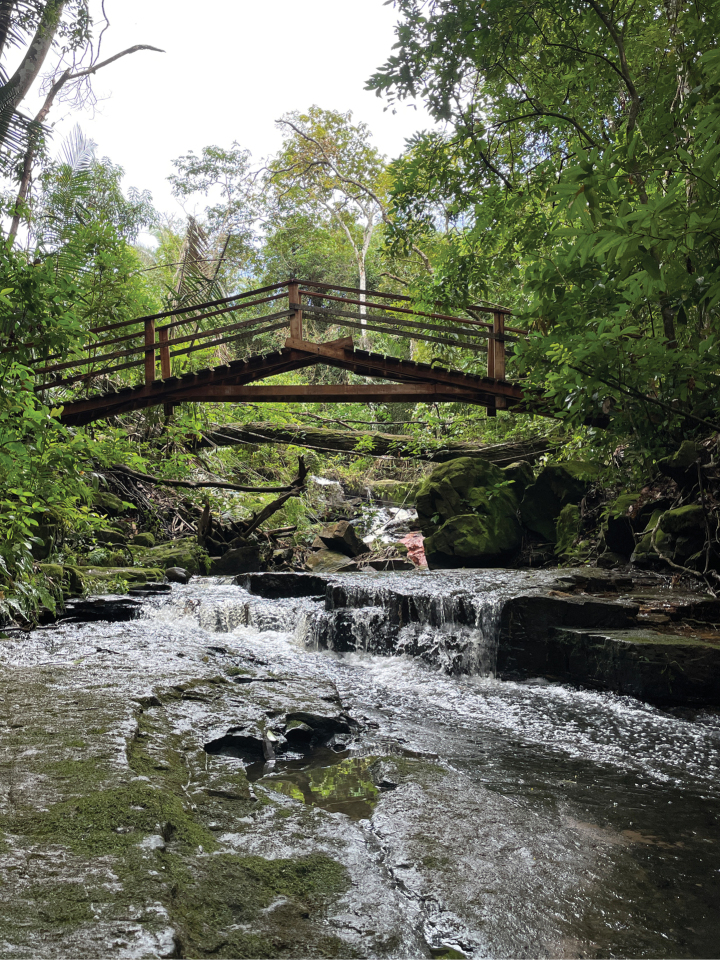
The type locality of *Poxyaibamberusubajarensis* Andersen & Dantas, sp. nov. in Ubajara National Park where the holotype was collected in a light trap near the Cafundó waterfall.

The species is known only from the type locality in the Ubajara National Park in northeastern Brazil. The park covers an area of 6.288 ha and receives abundant rainfall, averaging 1.400 mm annually, while temperatures typically range between 22 °C and 26 °C. The vegetation in the park is characterized by its high diversity, with higher elevations adorned by lush humid forests (Figueiredo 1997; Souza 1997). Lower elevations feature semi-deciduous tropical rainforest on sloping areas and arboreal Caatinga (thorny deciduous forest) in the lower regions. The specimens were collected using a light trap placed about 20 m upstream of a waterfall (Fig. 5) at an elevation of 805 m a.s.l. At the sampling time, water temperature was 21.5 °C, pH was 7.05, and electric conductivity was 137.6 µS/cm.

## ﻿Discussion

**Figure 6.**
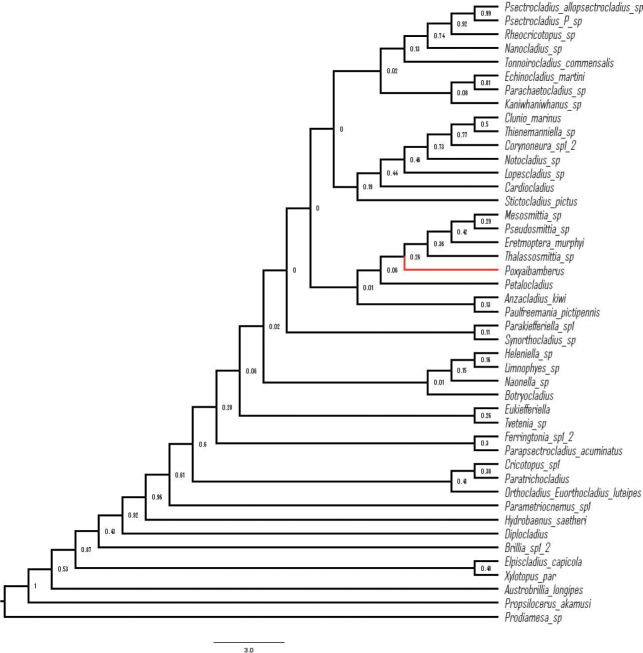
Position of *Poxyaibamberus* Andersen & Dantas, gen. nov. (marked in red) in the phylogenetic tree of Orthocladiinae, reconstructed with Bayesian inference (all posterior probabilities of the nodes are displayed, regardless of the value). This is a majority-rule consensus tree based on morphology only.

In a consensus tree produced in the Bayesian analysis neighboring search, *Poxyaibamberus* falls out with *Mesosmittia* Brundin, 1956, *Pseudosmittia* Goetghebuer, 1932, *Eretmoptera* Kellog, 1900, *Thalassosmittia* Strenzke & Remmert, 1957, and *Petalocladius* Sublette & Wirth, 1972 (Fig. 6). Both *Pseudosmittia*, with more than 100 species, and *Mesosmittia*, with 18 species, are distributed in most parts of the world (Ferrington and Sæther 2011; Ashe and O’Connor 2012). The Neotropical species have been treated by Andersen et al. (2010) and Andersen and Mendes (2002). However, a position close to *Thalassosmittia*, *Eretmoptera*, and *Petalocladius* is notable. *Thalassosmittia* has 11 named species distributed in the Afrotropical, Nearctic, Neotropical, and Palaearctic regions (Ashe and O’Connor 2012). With few exceptions, *Thalassosmittia* representatives are marine shore dwellers (Andersen et al. 2013). However, the only *Thalassosmittia* described from the Neotropical Region is *T.amazonica* Andersen & Pinho, 2014, which was collected in the Amazon rainforest near Manaus (Andersen and Pinho 2014). *Eretmoptera* is a genus with two named species that are distributed in the Nearctic Region and in Antarctica. The adults are wingless. *Eretmopterabrowni* Kellog, 1900 is distributed in California, while *E.murphyi* Schaeffer, 1914, was described from the island of South Georgia and is later introduced to Signy Island in the South Orkney Islands (Ashe and O’Connor 2012). The species is apparently parthenogenetic; the larva has a two-year life cycle and lives in damp moss and peat where they are thought to feed on decaying vegetation (Cranston 1985; Convey 1992). *Petalocladius*, with two included species, is distributed on the Caribbean Islands Jamaica and Hispaniola (Andersen et al. 2024). Both species have been collected in mountainous areas, and *P.dominiensis* Andersen & Baranov, 2024 was trapped close to a rather rapid, small river with rocky and stony substrates.

**Figure 7.**
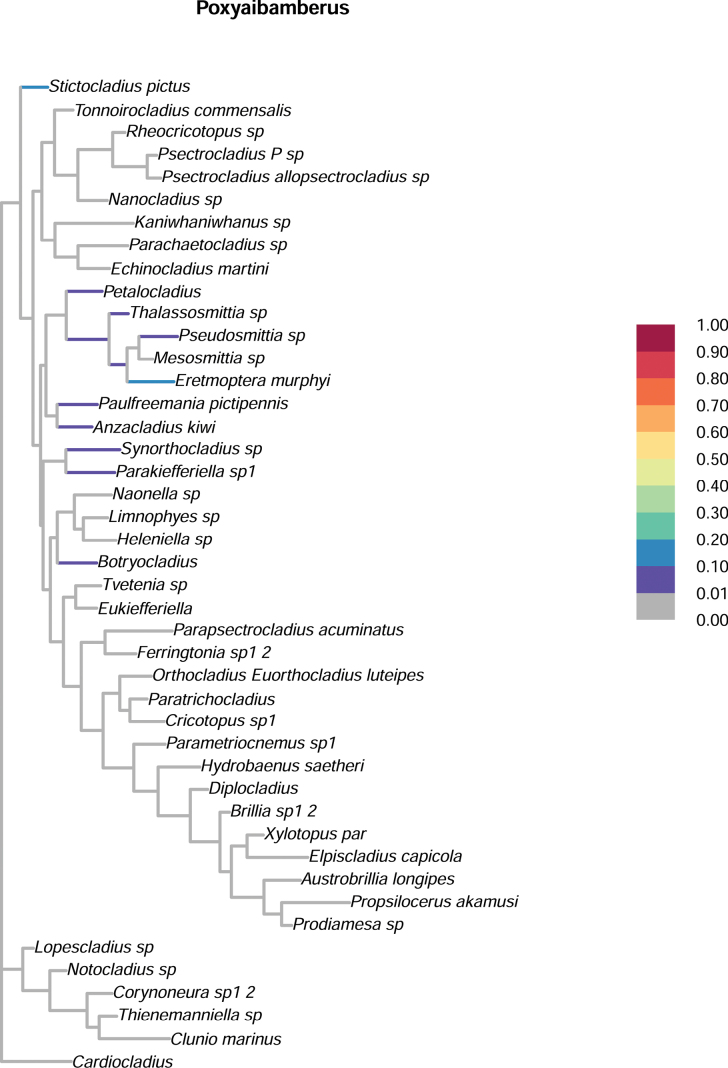
Rogue plot of the frequency of placement of *Poxyaibamberus* Andersen & Dantas, gen. nov. in the Bayesian tree from Fig. 6, based on the frequency of occurrence of *Poxyaibamberus* at a given node, based on the 50,001 most congruent trees from the Bayesian analysis of the genus’s morphology.

When posterior position probability mapping (“Rogue plots”) was applied, *Poxyaibamberus* was plotted in approximately 20% of the cases next to *Stictocladius* Edwards, 1931 and in approximately 20% of the cases next to *Pseudosmittia* / *Eretmoptera* (Fig. 7). While these were the most frequent positions within the generated trees, it plots near *Eretmoptera* in the consensus tree due to cumulatively more frequent occurrence (ca 40%) in or near this clade (Fig. 6). The position of *Stictocladius* in relation to *Poxyaibamberus* remains highly uncertain due to the lack of knowledge of the immature stages of the new genus. It is difficult to elaborate on the character distribution in the tree, as we have used an mcmc-based Bayesian approach, rather than maximum parsimony, which precludes more detailed analysis of the characters distribution. We have used the Bayesian approach, rather than maximum parsimony because of a deficiency in knowledge of many characters in the new genus. Thus, a more detailed analysis of the character distribution and sister relationships of *Poxyaibamberus* will have to wait until description of the additional life stages of the genus.

## Supplementary Material

XML Treatment for Poxyaibamberus

XML Treatment for Poxyaibamberus jamanximensis

XML Treatment for Poxyaibamberus ubajarensis

## ﻿Additional information

### Author ORCIDs

Trond Andersen https://orcid.org/0000-0003-2201-1870

Galileu P. S. Dantas https://orcid.org/0000-0002-9155-533X

Viktor Baranov https://orcid.org/0000-0003-1893-3215

Annui M. Sanz-laParra https://orcid.org/0000-0003-0580-6890

Humberto F. Mendes https://orcid.org/0000-0002-8261-3665

Neusa Hamada https://orcid.org/0000-0002-3526-5426

### Data availability

All of the data that support the findings of this study are available in the main text or in https://github.com/chironomus/Poxyaibamberus-.
